# Can meaning make cents? Making the meaning of work salient for US manufacturing workers

**DOI:** 10.1371/journal.pone.0265590

**Published:** 2022-07-08

**Authors:** Alberto Salamone, Grace Lordan

**Affiliations:** Department of Psychological and Behavioural Science, The London School of Economics and Political Science, London, United Kingdom; Xiamen University, CHINA

## Abstract

We conducted a field experiment in a small electronics manufacturing firm in the US with the specific aim to improve minutes worked, punctuality, tardiness and safety checks. Our intervention was to put posters on the production floor on a random day, which made salient to the blue-collar employees the meaning and importance of their job, which comprised of routine repetitive tasks, in a before and after design. Overall, the intervention was a success with positive and significant effects consistently found for the outcomes both immediately after the experiment finished (+3 days) and also more than two weeks after (+15 days). Our study highlights it is possible to motivate blue collar manual workers intrinsically by drawing attention to the meaning of their work.

## Introduction

Specific to manufacturing, competition within the industry is fierce and margins are tight, making it difficult to motivate workers through higher pay. Simultaneously, manufacturing workers are incredibly important to the US economy, given that the industry itself has never had a persistent decline in output, but rather become more capital intensive. Today the manufacturing sector’s output is 5% higher than it was in 2000 [1]. Given the existence of hard budget constraints making it difficult to increase pay, it is valuable to explore whether harnessing intrinsic motivation can increase the productivity of blue-collar routine workers in manufacturing. Specifically, we explore whether a poster intervention on a production floor in an electronics manufacturing firm, which makes salient to the blue-collar employees the meaning and importance of their routine job, can have a positive impact on a number of outcomes that are important to the firm. We specifically chose an electronics firm for this experiment given the importance of this area of manufacturing in driving US manufacturing output growth in the last two decades [2].

To our knowledge we are the first study to consider harnessing the intrinsic meaning of work in an experimental field setting for blue collar workers in manufacturing. The industry pressures described above bellies the importance of our work for both the academic literature and also firm level policies that aim to entice workers to work longer and better. Notably, research that does explore meaning at work is mainly related to non-manual or service workers, and produces associations rather than causal effects. However, these associations crisscross many domains that management care about, suggesting positive relationships between increasing meaningfulness with work engagement [3], job satisfaction [4], stress reduction [5–7], individual performance [8, 9], personal fulfillment [10], absenteeism [11], and overall work motivation [12]. Of particular note are papers by Grant [13] and Grant [14], who, in an experimental field setting, demonstrated that by highlighting the benefits of their output to fundraisers for educational scholarships the workers made more calls and the donations received increased significantly. Also related to our work is Chandler and Kapelner [15], who recruited 2500 people with MTurk and randomly created three groups: the meaningful group (to whom it was told that their work was needed to study tumoral cells), the control group (to whom no information was given) and the shredded condition (to whom it was told that the data entered would not be looked at all). The authors found that the group in the meaningful condition performed the best overall, whereas the one in the shredded condition performed significantly worse.

To allow us empirically test whether harnessing meaning at work can improve outcomes in a blue-collar manufacturing firm, we collaborated with a small and medium sized enterprise (SME) to run a field experiment. The SME is a US owned electronic manufacturing service industry, who employees 39 people on their production floor. Although a small number of workers, we are able to gather outcome data on a daily basis making it possible to plausibly estimate the effects of our intervention in a before and after framework, both in terms of the timing of the effects and also the estimates.

Individuals in our study are tasked with producing electronic boards. These workers earn between $12 and $15 per hour. The tasks executed by the workers are repetitive, each of them performing only one specific task. These workers have never seen the final products of their work, and so the meaning of these tasks is not obvious to them. The aim of our intervention is to make the overall meaning of their tasks salient. Finding meaning in one’s activities has been linked to survival [16], happiness [17], and motivation [18]. It has also been shown to be beneficial to workers in terms of reduction in stress, cynicism, satisfaction and sense of fulfilment [9, 10]. Wrzesniewski et al. [19] suggest that individuals can define their work as a job, a career or a calling. For those who define their work as a job, they see their work only as a means to pay their bills. Extrinsic motivation is then the main reason for their work. Those who define their work as a career, besides from their paycheck, are motivated by career advancement and finding meaning in what they do. However, we argue that there is potential for those who define their work as a job to also find meaning in what they do beyond career advancement. We contribute by exploring this hypothesis for a group of workers whose meaning in their work is not initially obvious (as compared to other low skilled work such as caring for the elderly or working as fundraisers to increase donations for a good cause). We note here an innovative lab experiment by Ariely [20] who also highlight that destroying Lego models caused participants to lose moral and be significantly less productive when asked to build additional models as compared to a control group who got to see their work remain standing- specifically they built 40% more models. For the workers we are studying, the tasks are repetitive and the final application of what they have contributed to is not known. These jobs require a high psychological demand and the worker’s autonomy is low [21].

Our intervention places posters that make the meaning of these jobs salient on the production floor on a random day. Overall, this intervention was a success. We find that when exposed to the poster intervention the workers work longer, are more punctual and are less likely to be absent. In addition, we find they are significantly more likely to adhere to safety checks. We find some evidence of adaptation over time to the intervention; however, the process is slow and there are still significant effects more than two weeks after the posters were placed on the production floor.

### Context

In our study we observe two worker types: 32 electronic assemblers and 7 electronic testers. These workers begin their day at 7 am and finish at 3.30pm. In between they have two 12-minute breaks (9 am and 2 pm), as well as a 30-minute lunch break at 11.30 am. The electronics assemblers spend their time pre-bending, mounting and hand-soldering electronic components onto an electronic board. Specifically, pre-bending consists of taking the two pins of each component and one by one giving them a precise shape that will fit two holes in the electronic board where it needs to be soldered. The pre-bend needs to be accurate and the shape and length of the pins need to be compliant with the design specifications. After the pre-bend phase, the components are then mounted manually onto the electronics board. The two pins are inserted into two holes and are then cut on the other side of the board leaving a small hook. The component is then hand soldered using a solder tool which has the shape of a pen with the tip that reaches a temperature that melts metal. The assembler holds the soldering tool in one hand and a piece of solid lead wire in the other. The solder occurs when the metal wire meets the hot tip of the pen exactly on the extremity of the metal pin. All three phases need to follow specific standards which are described and defined by international worldwide recognized standards for the Institute for Printed Circuits. To maximize efficiency, assemblers are divided into three groups, each of them performing the same task all day long.

Testers spend their time inspecting, polishing and testing the electronic boards built. On each electronics board there are hundreds of components and hundreds of pins that have been soldered. Each visual inspection requires maximum attention to make sure that the boards are compliant with the necessary standards and considered ready to be tested. The testers in this phase follow the specifications of the standard to assess the quality of each board and decide whether the board is functional or needs to be reworked. The second activity is testing. The board is inserted in a slot that connects the board to the tester and the testing phase is launched. There are several steps of the test in which the tester is required to push some buttons dictated by the test program and needs to put the test points to specific areas of the board. While the company makes many different board types, to maximize efficiency each tester is usually assigned the same board type. This makes the work of the testers repetitive. Workers do not receive orientation training at hire. They join their team and get on-the-job training.

### The intervention

We collaborated with a privately held company in South East USA operating in the Electronics Manufacturing Sector. The company employees 39 manual workers on the production floor and builds electronics boards. The employees were unaware of the intervention, and consent was given to place the posters by the company. These employees are comprised of 27 females and 12 males of which 15 are Black and 24 are White. Further, 13 of these workers were millennials (less than 38 years) when this experiment was run. As discussed, the main tasks are routine and repetitive, involving mounting electronics components on a board, hand soldering components, testing and inspecting boards. The workers do not see the end application and earn between $12 and $15 per hour. In order to make the meaning of work salient we created posters that would draw attention to how important their end use product is, as well as the workers’ own skills in creating this product.

The use of motivational posters in a firm setting is not new. For example, Vora et al., [22], used posters to encourage employees to take the stairs over the elevator while at work, and Zainab [23] used a poster intervention to encourage healthier eating alternatives while at work. However, we know of no study to draw on posters to make the meaning of work salient for any worker type.

When designing the posters, we avoided any communication about the company’s own values, objectives, or any reference to its leadership so as to avoid igniting a dissonance between the employee’s feelings for their company and the message of meaning in the poster [24, 25]. Rather, the posters we designed focused only on the personal skills of the employees, and how those skills contribute to the realization of a final product that has great value to society. The posters were hung on walls in very visible places on the production floor. We observe data for 21 working days ex ante and 19 working days ex post. These data relate to Monday through Friday.

The posters were hung at a time when no employees were at work, and no mention was made to the employees of their presence by management. This allowed the employees to interpret the messages of the posters without interference by other parties. Workers on the production floor were never told explicitly of the experiment and no explanation was given as to the reasons of the posters. Only the human resource manager and the general manager, who helped with the setting up of the experiment and the data collection, were aware of the study. We note that the human resources manager and the general manager both sit in an office away from the production floor and were not in contact with workers on the floor during the experiment.

**Fig 1:**
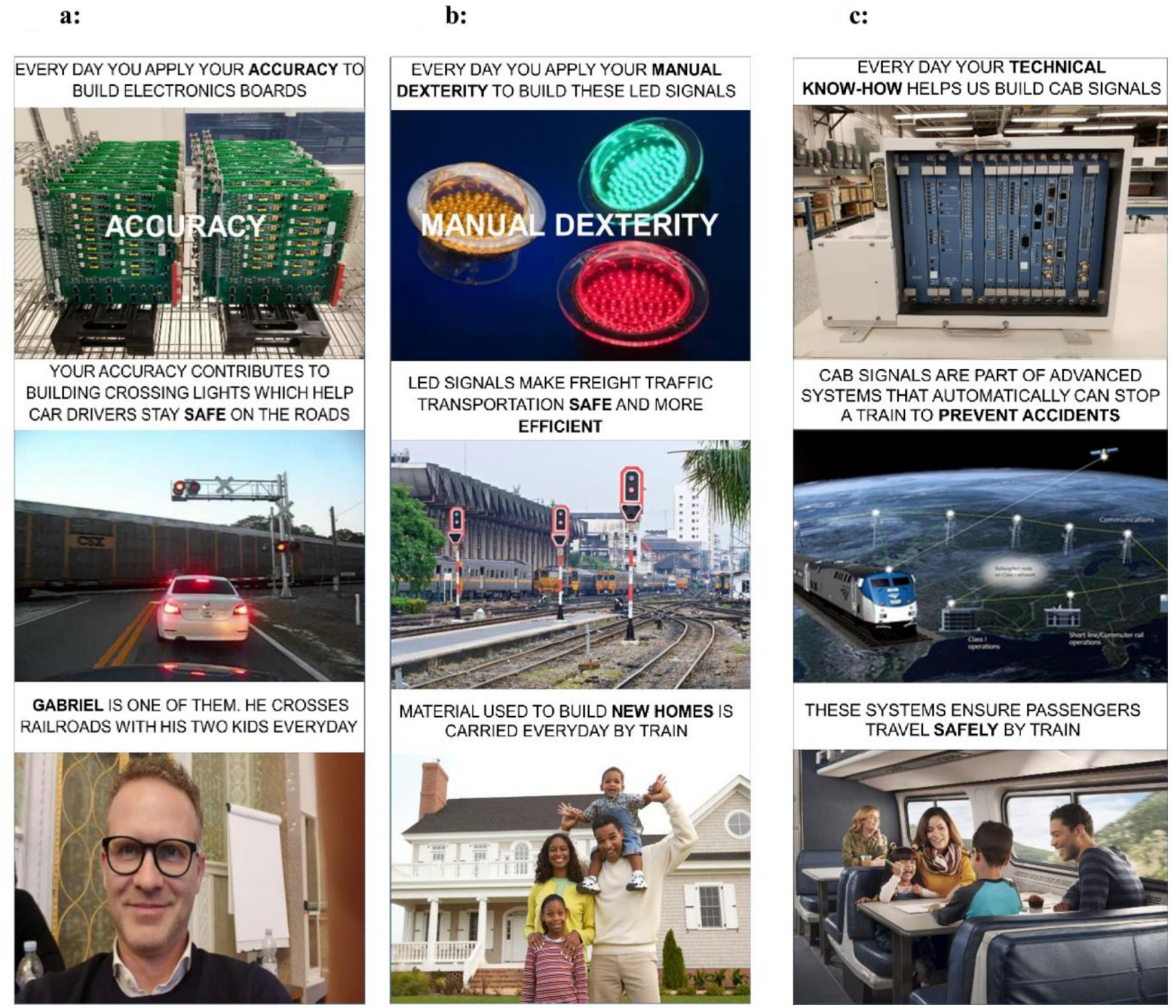
(a, b, c) The three types of posters that were used for the intervention.

In total there were nine posters with three distinct designs: the first poster is in Fig 1a, composed of three different pictures connecting the skills of the workers with the final product and finally with the greater good for the society. For instance, in Fig 1a we make salient that Accuracy contributes to building crossing lights, which in turn keeps Gabriel safe. Similarly for the three pictures in the second poster shown in Fig 1b, and the three pictures in the third poster shown in Fig 1c.

Specifically, each poster was designed with three distinct illustrations that have an accompanying text.

The first component of each of the three poster designs specified a specific skill that the worker drew on constantly each day, accompanied by a picture of the electronic boards that workers contribute to build. The intention of this first component is to tap into authenticity and self-efficacy as mechanisms to increase the meaning of work. Authenticity through giving the workers a realization of the skills they are employing every day, and the alignment of these skills with the work they are doing. Self-efficacy, through increasing their self-awareness of their own skills, as well as their personal worth, while at work. To define the skills, we turn to the ONET data (Version 15) on skills for persons who are team assemblers and electronics testers. These production associate roles have job codes 51–2092 and 51–9061 respectively and represent the closest occupation matches to the jobs being currently done on our collaborator’s production floor.

Specifically, a team assembler

“*Works as part of a team having responsibility for assembling an entire product or component of a product*. *Team assemblers can perform all tasks conducted by the team in the assembly process and rotate through all or most of them rather than being assigned to a specific task on a permanent basis*. *May participate in making management decisions affecting the work*. *Includes team leaders who work as part of the team”*

and an electronics tester

*“Inspects*, *tests*, *sorts*, *samples*, *or weighs non-agricultural raw materials or processed*, *machined*, *fabricated*, *or assembled parts or products for defects*, *wear*, *and deviations from specifications*. *They may use precision measuring instruments and complex test equipment*.*”*

We chose three distinct and specialised skills from the ONET database that are highly associated with these jobs, which feature on our posters 1a through 1c. These are i) accuracy ii) manual dexterity and iii) technical know-how.

The second component of each poster is an illustration and text description of the final product of the employee’s work. These final products are crossing lights, LED signals for freight traffic and CAB systems (Fig 1a–1c respectively) that form part of technology that allows trains to automatically stop if needed. The aim of this component of the poster is to tap into the workers overall sense of purpose. Sense of purpose is enhanced by explaining the end application and the *raison-d’être* of the product. These workers enhance safety which has clear value to society.

The third component of each poster contains a specific text description of the potential benefits that the workers’ final product brings to society. This component emphasizes the greater good for society from the perspective. This component draws on the power of narrative, given people pay more attention to a single story over statistics [26]. Specifically, the last component of each poster highlights a person who is benefiting from the final product. In Fig 1a this is Gabriel, a middle-aged man who relies on safe railroads to get his kids to their destination each day. In Fig 1b, a happy family stands outside the house that was built with materials that a train transported safely to their destination. Finally, in Fig 1c, a family and a single female are relaxed because they are travelling safely on a train journey.

Overall, the posters aim to make the link between the specific skills the workers possess (recognition) with the final application (purpose) and finally the greater good for society (transcendence) salient. To take Fig 1a as an illustrative example:

1. The first component of the poster invokes recognition through the text “*Every day you apply your accuracy to build electronics boards”*. We are drawing attention to accuracy as a valuable skill possessed by the workers on the production floor.
2. The second component of the poster invokes purpose through the text: “*Your accuracy contributes to building crossing lights which help car drivers stay safe on the roads”*. In this case we make salient that safety is the overall purpose of the product being built by the workers.
3. The third component of the poster aims to make transcendence more salient through the text “*Gabriel is one of them*. *He crosses railroads with his two kids every day”*. This highlights that Gabriel and his children are three people benefiting from the product and staying safe thanks to the accuracy of the workers. Visually showing Gabriel helps close the social distance between him and the workers.

### An intervention that makes meaning salient

Our intervention aims to make salient to the employees we are studying what the value of their work is through a series of posters. The idea that outcomes of the firm may improve if the meaning of work is made salient aligns well with many conceptual frameworks that have been introduced to allow management scholars evaluate the benefits of meaningful work in an organizational setting. For example, Cameron [10] defines work as meaningful if some or all of the following four attributes are present i) there is an obvious impact on humanity ii) it aligns with a worker’s personal values iii) it creates positive ripple effects that go beyond the immediate benefits generated and iv) it builds a sense of community. Our intervention speaks directly to i). Specifically, by making the end uses of the product salient we are drawing attention to the fact that the employee’s work forms part of products which keep people safe as they go about their lives.

In a separate framework, Rosso et al. [9] argue that there are seven mechanisms scholars have recognized to be effective to increase meaning. These are: authenticity, as the alignment between the true self and one’s behavior; self-efficacy, as the self-awareness of one’s abilities; self-esteem, as the evaluation of one’s self-worth; purpose, as a sense of intentionality in life; belongingness, as the drive to feel part of a community; transcendence, as the connection of oneself to a greater entity; and cultural sense-making, as the role of the cultural context on the construction of meaning. The posters designed for this work have the potential to reach authenticity, self-efficacy and self-esteem.

The perception of meaning can also be enhanced by transformational leadership [24, 25]and by job design [27]. The former is how leaders behave in charismatic ways (*idealized influence*), inspire with an appealing vision (*inspirational motivation*), challenge assumptions (*intellectual stimulation*), and coach their followers (*individualized consideration*). The latter is how the job is designed and how the five job characteristics, variety, identity, significance, autonomy, feedback, are made explicit in the job design [8]. Grant [14] added a third dimension as a way to enhance the perception of meaning: putting the beneficiaries of the products or services the employees contribute to building in direct contact with the employees. Our posters rely on this domain as a mechanism through which to increase the meaning of work for the employees we are studying.

## Methods

Our intervention had a before and after design. In essence, 39 people experience work as usual, we observe their daily data, and there is a random change to their environment at the beginning of week five. Everyone is a complier, in the sense that they cannot control their exposure to the posters. We cannot know how much attention each person paid to the poster so calculating a dose response to exposure to the posters is not possible.

We note that it is plausible that we can assess the causal impact of the posters as the before and after period are stable. First, the time lag between the before and after is short, with daily data available for us to analysis. Second, one of the authors had direct contact with the firm throughout the experiment, allowing us have a closer relationship with the organization that is otherwise usual in research. We can say with certainty there were no particular events (e.g. weather, political, local or within the company) or any other special communications over the four-week period when the posters were on display. Nor was there any change in demand between before and during the intervention. In both periods, the company was behind with production and therefore welcoming as much overtime as possible from workers to increase output. Employees were already doing paid overtime before the intervention to a stable and constant rate. No communication about the need for more overtime was communicated to the workers along the whole period of the experiment. Any additional over time done during the intervention could not therefore be attributed to the extrinsic motivation of getting paid just because overtime was allowed, done, and paid before and during the intervention.

Therefore, we estimate:

$$Y_{i}=\alpha_{\text{o}}+\beta_{1}treatment_{t}+{\text{D}}_{\text{d}}+\varepsilon_{i}$$ (1)

In Eq 1 *Y* is an outcome of interest (see below) and *treatment = 1* on the day the poster intervention started and from then onwards. It is zero otherwise. D_d_ are a set of five dummy variables that control for each weekday. We have four weeks of data ex ante and ex post, and have confidence that our intervention was the only one that could alter outcomes in the company we collaborated with. Therefore, in our main specification we use the entire sample available ex ante. We do note that narrowing the sample to 10 days (rather than 17 days) does not affect our results.

We present estimates that vary the quantity of data used ex post. This allows us to consider whether effects changed–and whether there was adaptation to the intervention—over time. Specifically, we document estimates that include data +3, +6, +9, +12, and +15 days ex-post. All models are estimates by ordinary least squares and standard errors are clustered by worker.

Of course, there may be a tendency for variations in exposure, and also heterogenous treatment effects, across individuals. Because we collect daily data on employees, we can allow for this possibility by including individual fixed effects in our regression analysis. In a second set of analyses, we consider this alternate specification, with identification of effects coming from the average of the within individual response to our intervention. We therefore estimate:

$$Y_{it}=\alpha_{\text{i}}+\beta_{1}treatment_{it}+{\text{D}}_{\text{d}}+\varepsilon_{it}$$ (2)

### Outcome data

We are interested in linking the intervention to hard data which matters to the firm we collaborated with. Unfortunately, as each individual contributes to only one activity that makes the final product, daily production output cannot be measured at the individual level i.e. it is not separable by individual. Instead, we use the number of minutes worked per day as a proxy for productivity.

We note that more minutes worked is not necessarily an indicator of increased productivity, unless the efficiency of the extra minutes worked is the same or higher than the baseline. To check for this aspect, we compared the overall production output per minute worked before and during the treatment collectively. This exercise highlighted that there was a constant-returns to minutes worked across all of the study window. That is, each additional minute worked in the treatment window, over baseline, contributed to overall productivity with the same proportional gain. Minutes worked per day were collected daily by reading the clock-in and clock-out time per day for each worker. Lunch breaks are also recorded and omitted from minutes worked calculations. Both paid time off days and days in which workers requested hours off were also omitted. Our measure of minutes worked per day excludes individuals who are absent. We note, that there is no upper limit on the amount of overtime that can be done by these workers, as historically the firm faces on-time delivery pressures. The skilled manual nature of the routine tasks being done by these workers means that training costs act as a barrier to hiring casual labor so the firm relies on workers choosing to work longer hours. This message has long been communicated to the workers in our study by management. Overtime is paid per increase of each minute worked.

**Fig 2:**
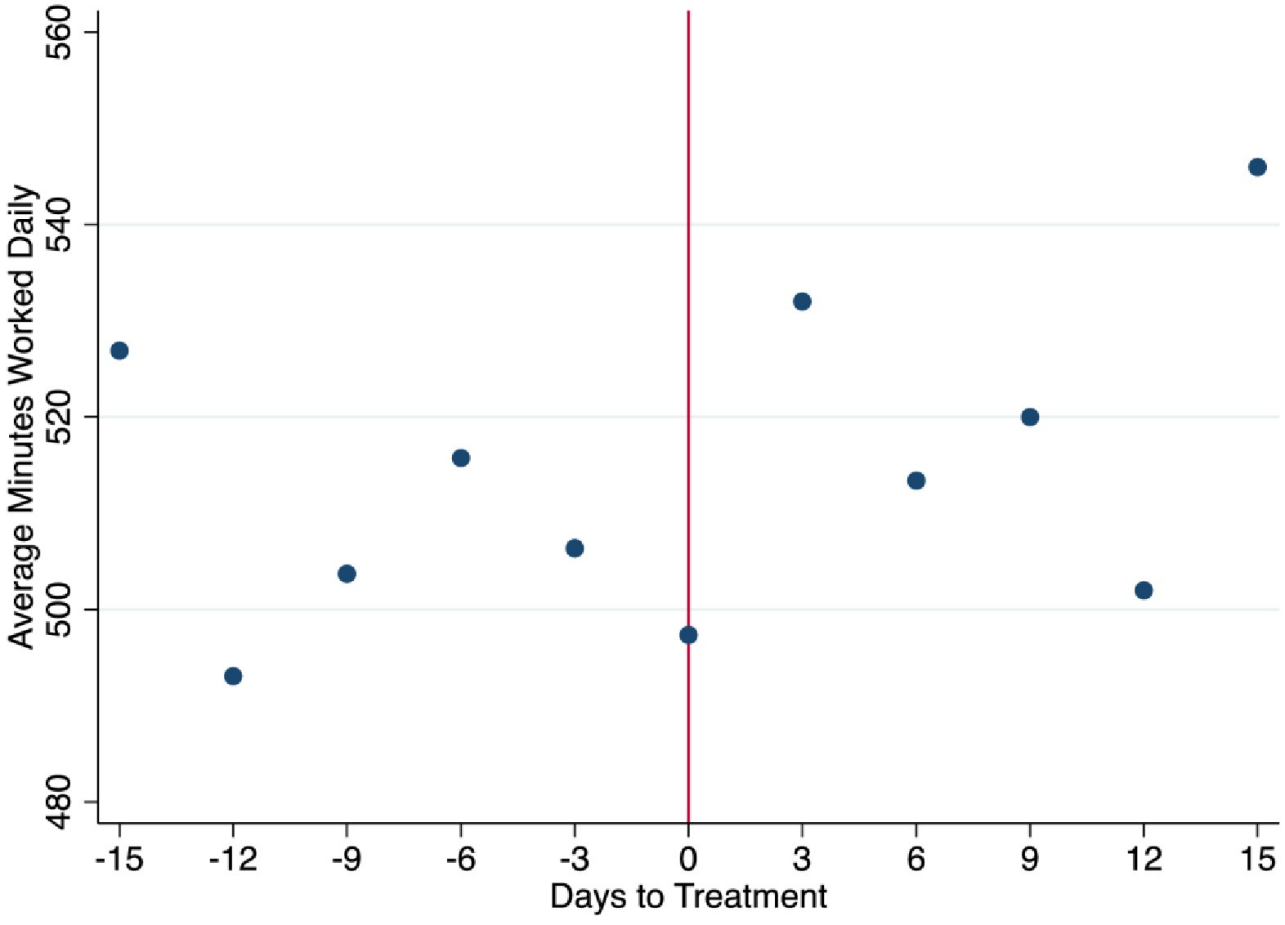
Ex post and ex ante average daily minutes worked.

Fig 2 illustrates daily data +3, +6, +9, +12 and +15 days after the poster intervention, in addition to the mirror daily data ex ante for average minutes worked per day. Specifically, we average data over individuals at this specific day. Overall, Fig 2 highlights a clear discontinuity in minutes worked +3 days after the poster interventions. This suggests that poster intervention did indeed produce an increase of the minutes worked of the workers.

**Fig 3:**
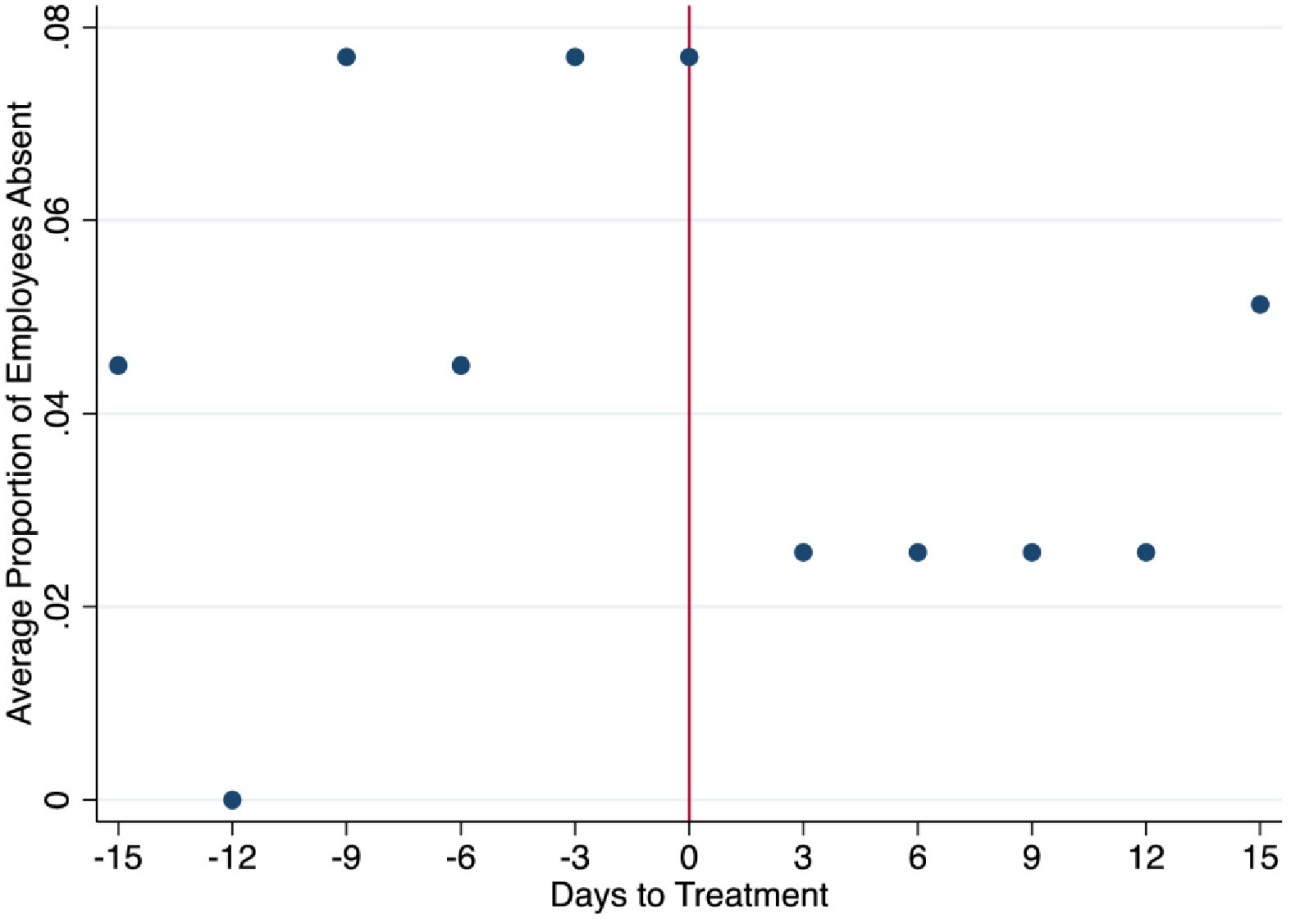
Ex post and ex ante proportion of employees absent.

We complement our measure of minutes worked with a second outcome that is assigned equal to 1 if a person is absent from work and zero otherwise. This allows us to speak to effects of the intervention on absenteeism. Paid and medical leaves are excluded and not counted in the absenteeism variable. Absenteeism measures the non-justified or non-planned absences. Workers who do not show up at work for no justified reason can be written up, suspended, or laid off if this becomes a recurrent event. Fig 3 illustrates average data for particular days ex post and ex ante the intervention, which are defined consistent with Fig 2. Overall, Fig 3 again highlights a clear discontinuity in absenteeism three days after the poster intervention was introduced, suggesting that the poster intervention lowered the likelihood of not turning up for work.

**Fig 4:**
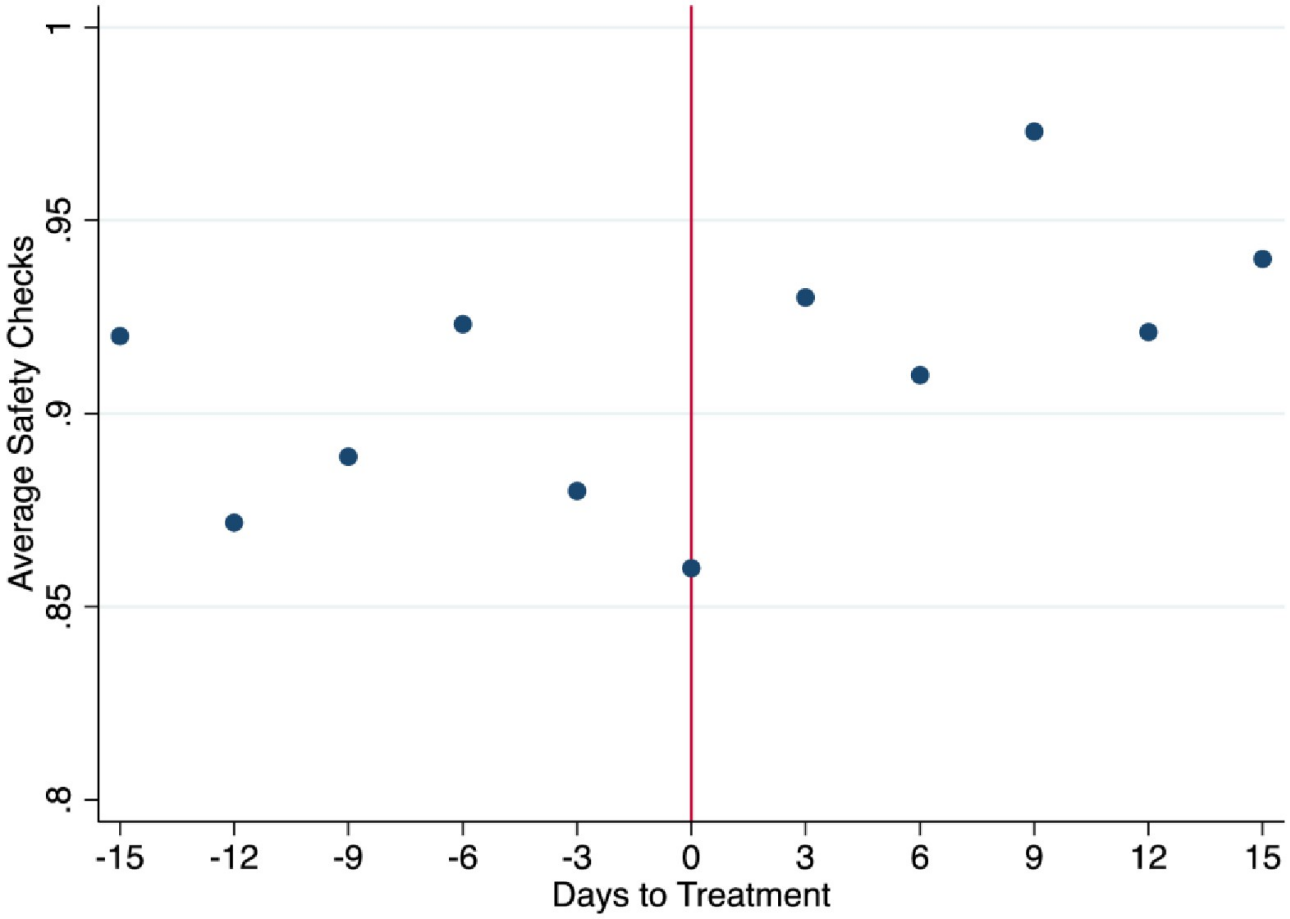
Before and after averages of safety checks.

We also examine the percentage of compliance of workers to ESD (Electrostatic Discharge) safety checks. Electrostatic Discharge is a charge that human bodies accumulate naturally, and when they touch something, the charge suddenly moves causing an electric shock to the object touched. If no precautions against ESD are taken, operators can unwittily destroy or weaken the electronic boards they are manufacturing while producing them, causing significant losses in output for the company if caught immediately, or a faulty product for a customer downstream. In order to avoid these failures, every morning workers wear the ESD equipment which consists of a ESD jacket and a ESD wrist and go to a machine to check whether their equipment is fully functional and is correctly preventing the worker from accumulating any electrical charges, which could be lethal for the electronic components. Workers may fail to do the check simply because they forget to go to the machine and perform the test. Management constantly sends reminders about the importance of performing these checks. Workers who repeatedly fail to perform the check are warned and invited to pay attention. Management can intervene and write up the workers who persist. The goal of the company is to be close to 100%. This check is logged on a computer and the indicator defined measures daily the percentage of compliance of the operators. 100% means that all workers have checked that the ESD equipment has been worn correctly. The ESD check is a binary variable. Raw daily safety check data was collected by the process engineer and given to the researchers. Fig 4 highlights a trend of a higher rate of safety checks after the poster intervention was introduced. This suggests the intervention was beneficial in getting workers to be more likely to complete safety checks.

**Fig 5:**
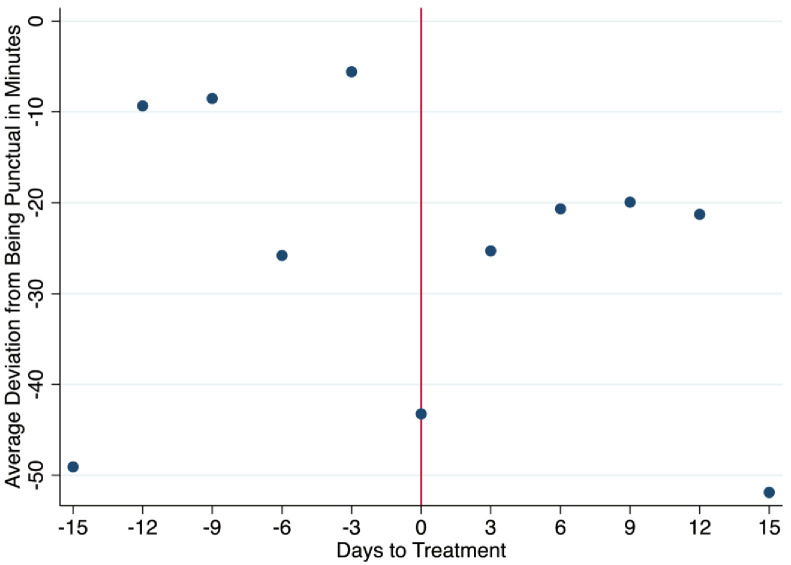
Before and after averages of punctuality.

**Fig 6:**
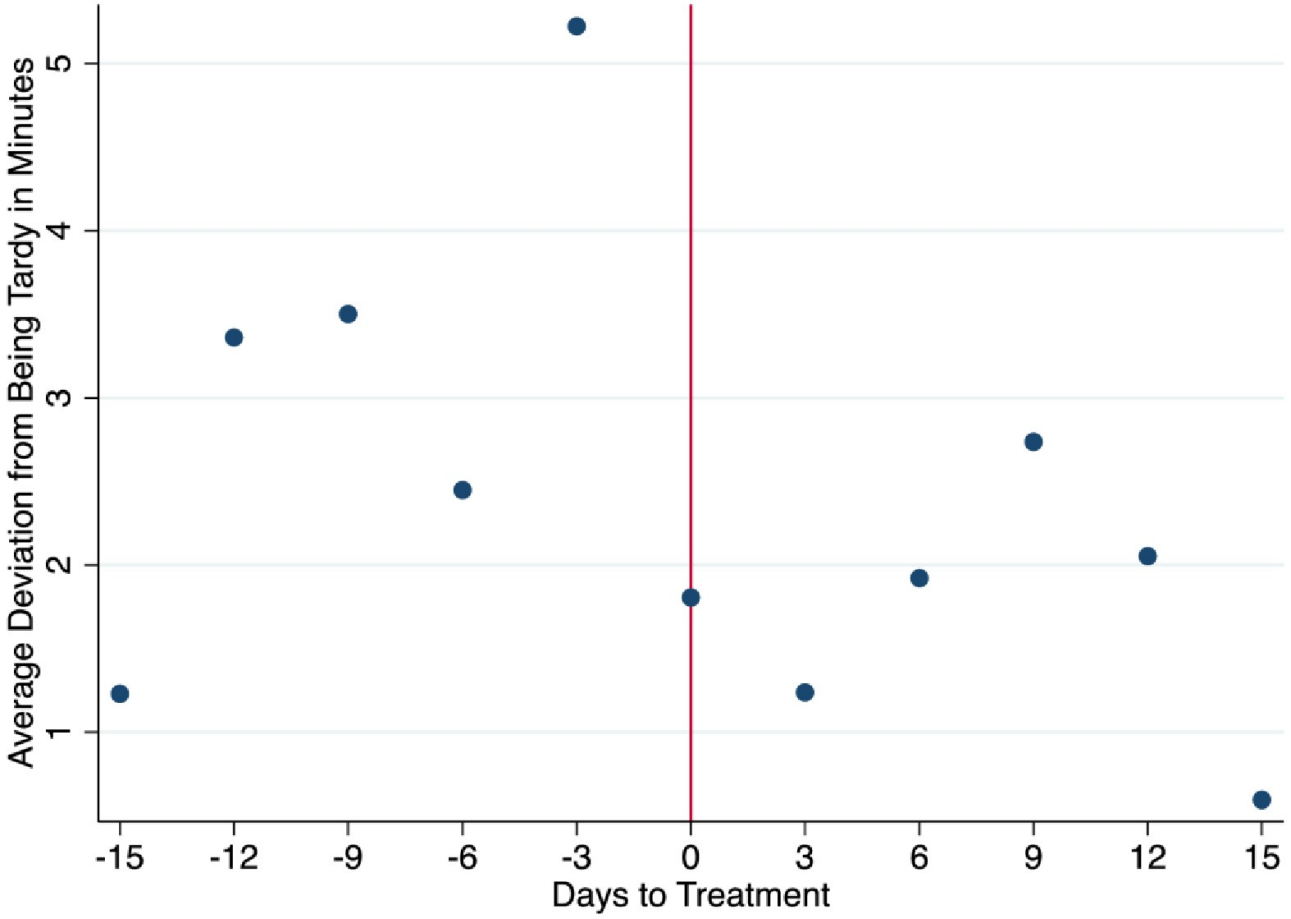
Before and after averages of tardiness.

We also consider daily punctuality. We view daily punctuality as a proxy of the level of engagement and willingness to comply with the written punctuality rules of the firm. Specifically, each morning workers are asked to arrive any time before 7 am. If workers keep coming late, they can be written up, suspended or laid off, depending on the gravity and the frequency. A worker is allowed to clock in before 7 am. The outcome is defined in minutes and is negative if the worker arrived earlier and positive if they arrived late. We also consider an additional definition of punctuality defined only as minutes of tardiness and recording as zero anyone who arrives early or on time. We call this variable tardiness. From Figs 5 and 6, looking at punctuality and tardiness, there is a visible change towards punctuality, and working overtime after the event, in addition to a shift away from being tardy. We note that the data on minutes worked, and punctuality were collected by the human resources manager and handed over to the researcher for analysis.

## Results

Table 1 documents the estimates pertaining to Eq 1. A number of stylised facts emerge. First, across all outcomes the signs on the coefficients are as expected and mostly significant. Second, over time there is no clear evidence of adaptation. That is, the coefficient does not obviously attenuate with the passage of days. Notably, after fifteen days almost all outcomes are still augmented significantly by the intervention. For example, excluding tardiness and punctuality, the average effect of the treatment is that workers work about 16 minutes more each day. This is a clear decrease in absence caused by the intervention of 16 percentage points. This level of a reduction in absenteeism implies an average decrease of around 0.25 days over the 15-day period. The effect on tardiness and punctuality are not significant but have the expected negative sign. Finally, the intervention improved the probability of safety checks by 5 percentage points.

**Table 1:** Main regression estimates.

	Minutes Worked Exclude Absence	Absence	Tardiness	Punctuality	Safety Checks
+3 Days	17.927**	-0.141***	-1.060	2.107	0.088***
	(7.470)	(0.051)	(1.877)	(3.640)	(0.023)
N	381	468	381	381	375
	Minutes Worked Exclude Absence	Absence	Tardiness	Punctuality	Safety Checks
+6 Days	20.080***	-0.175***	-2.036	-2.603	0.075***
	(5.049)	(0.034)	(1.249)	(2.811)	(0.019)
N	494	585	494	494	487
	Minutes Worked Exclude Absence	Absence	Tardiness	Punctuality	Safety Checks
+9 Days	18.379***	-0.168***	-1.821*	-3.953	0.080***
	(3.564)	(0.030)	(1.056)	(2.558)	(0.019)
N	608	702	608	608	599
	Minutes Worked Exclude Absence	Absence	Tardiness	Punctuality	Safety Checks
+12 Days	19.400***	-0.160***	-1.550*	-4.451*	0.068***
	(3.399)	(0.029)	(0.855)	(2.388)	(0.019)
N	720	819	720	720	707
	Minutes Worked Exclude Absence	Absence	Tardiness	Punctuality	Safety Checks
+15 Days	16.284***	-0.156***	-1.257	-0.528	0.059***
	(5.101)	(0.028)	(0.859)	(2.319)	(0.018)
N	832	936	832	832	818

Notes: Standard errors are in brackets and are clustered at the worker level.

*, **, and *** denote significance at the 10%, 5% and 1% levels respectively.

We also control for days of the week dummies.

Table 2 documents the estimates pertaining to Eq 2. When we include individual fixed effects in the regressions the estimates do change modestly. This implies a potential for heterogenous treatment effects by some trait that is fixed at the individual level. Notably, all effects are attenuated only very slightly as compared to Table 1.

**Table 2:** Fixed effects estimates.

	Minutes Worked Exclude Absence	Absence	Tardiness	Punctuality	Safety Checks
+3 Days	16.933**	-0.137**	-1.325	0.858	0.089***
	(7.627)	(0.054)	(2.003)	(3.759)	(0.026)
N	381	468	381	381	375
	Minutes Worked Exclude Absence	Absence	Tardiness	Punctuality	Safety Checks
+6 Days	18.592***	-0.174***	-2.074	-2.876	0.074***
	(4.549)	(0.035)	(1.264)	(2.815)	(0.020)
N	494	585	494	494	487
	Minutes Worked Exclude Absence	Absence	Tardiness	Punctuality	Safety Checks
+9 Days	16.349***	-0.167***	-1.771	-3.989	0.078***
	(3.062)	(0.030)	(1.051)	(2.516)	(0.019)
N	608	702	608	608	599
	Minutes Worked Exclude Absence	Absence	Tardiness	Punctuality	Safety Checks
+12 Days	17.376***	-0.160***	-1.494*	-4.556*	0.066***
	(3.020)	(0.030)	(0.838)	(2.312)	(0.020)
N	720	819	720	720	707
	Minutes Worked Exclude Absence	Absence	Tardiness	Punctuality	Safety Checks
+15 Days	12.743***	-0.161***	-1.451	-4.266*	0.051***
	(4.563)	(0.029)	(0.867)	(2.197)	(0.019)
N	832	936	832	832	818

Notes: Standard errors are in brackets and are clustered at the worker level.

*, **, and *** denote significance at the 10%, 5% and 1% levels respectively.

We also control for days of the week dummies.

We are limited in the data that we have on workers so cannot fully probe the finding that there are heterogenous treatment effects. However, we do have data on race, gender and age. Therefore, we re-estimate Eq 2 and this time add each demographic interacted with the intervention. Specifically, for race we create a dummy that is assigned equal to 1 if the worker is Black and zero otherwise (all workers are either Black or White), and add an interaction between this dummy and the intervention to Eq 2. For gender we create a dummy that is assigned equal to 1 if the worker is female and zero otherwise and add an interaction between this female dummy and the intervention to Eq 2. Finally, we create a dummy that is equal to one if the worker is a millennial (less than 38 years when this experiment was run), and zero otherwise, along with an interaction between this dummy and the intervention. Results from theses analyses can be found in Table 3.

**Table 3:** Fixed effects estimates with interactions. (ex post +9 days).

	Minutes Worked Exclude Absence	Absence	Tardiness	Punctuality	Safety Checks
Treatment*Black	3.690	-0.040	-0.051	4.624	0.035
	(6.297)	(0.061)	(1.861)	(4.602)	(0.035)
Treatment	14.180***	-0.143***	-1.741*	-6.708***	0.057***
	(4.568)	(0.045)	(1.013)	(2.155)	(0.017)
N	608	702	608	608	599
	Minutes Worked Exclude Absence	Absence	Tardiness	Punctuality	Safety Checks
Treatment*Female	-8.638	-0.054	-4.080	0.566	-0.012
	(6.321)	(0.074)	(2.986)	(5.702)	(0.037)
Treatment	18.929***	-0.150***	-0.553	-4.158	0.082***
	(3.676)	(0.032)	(0.534)	(2.971)	(0.026)
N	608	702	608	608	599
	Minutes Worked Exclude Absence	Absence	Tardiness	Punctuality	Safety Checks
Treatment*Millennial	-8.219	-0.016	-4.205	-2.225	0.029
	(5.300)	(0.054)	(4.225)	(6.907)	(0.059)
Treatment	18.101***	-0.164***	-0.875*	-3.515	0.072***
	(3.765)	(0.037)	(0.473)	(2.701)	(0.019)
N	608	702	608	608	599

Notes: Standard errors are in brackets and are clustered at the worker level.

*, **, and *** denote significance at the 10%, 5% and 1% levels respectively.

We also control for days of the week dummies.

From Table 3 there is no real evidence of heterogenous treatment effects by race, gender or millennial status. In Table 4 we present estimates separately for our two worker types: Testers and Assemblers. The estimates in Table 4 highlight larger effects for Testers as compared to Assemblers for absence, tardiness and safety checkers. In contrast, there are greater impacts on punctuality for assemblers. However, overall, for both groups of workers, it would seem the poster intervention modified behaviour in a positive direction.

**Table 4:** Fixed effects estimates separately by worker type. (ex post +9 days).

	Minutes Worked Exclude Absence	Absence	Tardiness	Punctuality	Safety Checks
**Testers**	16.869	-0.298**	-2.772	-1.836	0.101*
	(10.050)	(0.089)	(1.667)	(3.315)	(0.044)
Treatment	16.869	-0.298**	-2.772	-1.836	0.101*
N	104	126	104	104	101
	Minutes Worked Exclude Absence	Absence	Tardiness	Punctuality	Safety Checks
**Assemblers**	16.347***	-0.138***	-1.574	-4.435	0.074***
	(3.193)	(0.030)	(1.226)	(2.979)	(0.022)
Treatment	16.347***	-0.138***	-1.574	-4.435	0.074***
N	504	576	504	504	498

Notes: Standard errors are in brackets and are clustered at the worker level.

*, **, and *** denote significance at the 10%, 5% and 1% levels respectively.

## Conclusions

We conducted a non-invasive field experiment in a SME manufacturing firm in the US with the specific aim to improve outcomes that are important to the firm. The outcomes that we studied are minutes worked, punctuality, tardiness, and safety checks. The SME employs 39 workers on their production floor. These workers are tasked with producing electronic boards and earn between $12 and $15 per hour. Although a small number of workers, we were able to gather outcome data on a daily basis making it possible to precisely estimate the causal effects of our intervention.

Our intervention was to put posters on the production floor on a random day, which made salient to the blue-collar employees the meaning and importance of their routine job tasks. This before and after design is credible, given that on a random day the workers environment was changed and there was nothing special about the days before or after the intervention. The workers we studied are employed in jobs that involve repetitive tasks, which to most people would be described as boring. In addition, for these workers the end product i.e. what the electronic boards are ultimately made into is not salient, so the point of their efforts is not obvious. We made the decision to have three parts to each poster. The first, was designed to invoke recognition of the workers own skill. The second, was designed to emphasize the purpose of the electronic boards (for example they end up creating crossing lights that keep people safe), and the third component to personalize the benefit of product (for example: we give the person who is being kept safe a face and an identity reducing social distance between the worker and the person depicted in the poster). To our knowledge, we are the first study to consider harnessing the intrinsic meaning of work in an experimental field setting for blue collar workers in manufacturing. In addition, we are the first study that we know of in any firm to utilize posters that make the meaning of work salient in an effort to improve firm outcomes.

We modeled the data retrieved from our field experiment to allow us study effects 3 days, 6 days, 9 days, 12 days and 15 days ex ante. Overall, the intervention is a success with positive and significant effects consistently found for minutes worked, punctuality and safety checks, both immediately after the experiment finished (+3 days) and also more than two weeks after (+15 days). Our estimates attenuate over time and suggest modest adaptation. However, after fifteen days there are still relatively large gains, bearing in mind this is a very cheap intervention ($9 per poster). For example, excluding absences, the average effect of the treatment is that workers work about 10 minutes more each day. Including absent workers this figure is about 75 minutes. We get similar conclusions (i.e., the intervention improves all but one of the outcomes we study) if we add fixed effects to our regressions, although the estimates do change suggesting that there are heterogenous effects of the treatment that vary by some aspect of the workers which is a fixed trait. We explore whether this fixed trait is age, gender or race and conclude that it is not. We do not have any information on other traits that were fixed to the individual to allow us to explore this further. In addition, we cannot know with certainty what aspect of the presence of the poster triggered the change in behaviour. However, our work does underline the importance of exploring this in the future to the extent we may be able to learn more about the possibilities of personalising interventions that are low cost, like the one we studied here.

Overall, we view our work as a first pass which shows it is possible to motivate blue collar manual workers intrinsically by drawing attention to the meaning of their work. It is limited by the small size of the company and its workforce, however the results suggest that cost efficient simple behavioural nudges, like our posters, can be used to motivate workers. In an industry where competition is fierce and margins are tight, we offer another alternative over extrinsic rewards which are the normal incentive touted for these worker types. We hope that this study encourages further research on blue collar manual workers to see if it is replicable, probes its external validity, and also encourages managers of other manufacturing firms to partner with academics in similar endeavors. We also hope that this work encourages managers, both within manufacturing and beyond, to consider the human aspect when managing blue collar workers and realize that focusing on people’s sense of purpose which is connected with their well-being, produces positive outcomes for the company. Future research may also capture self-reported work wellbeing measures such as job satisfaction, as anecdotal evidence from this particular firm suggested that our simple poster intervention also caused increases in happiness on the job. Such data is not routinely gathered in the firm we worked with, and we made a conscious decision not to gather it on this occasion so as to not draw attention to the fact that the posters represented an intervention that was being evaluated.
